# Breast cancer policy in Latin America: account of achievements and challenges in five countries

**DOI:** 10.1186/s12992-016-0177-5

**Published:** 2016-07-12

**Authors:** Gustavo Nigenda, Maria Cecilia Gonzalez-Robledo, Luz Maria Gonzalez-Robledo, Rosa Maria Bejarano-Arias

**Affiliations:** School of Medicine, Morelos State Autonomous University, Calle Leñeros esquina Iztaccíhuatl s/n Col. Volcanes, Cuernavaca, Morelos CP 62350 Mexico; Centre for Health Systems Research, National Institute of Public Health, Av. Universidad 655, Santa María A, Cuernavaca, Morelos CP 62100 Mexico; Health Science and Medicine School, Valley of Mexico University, Calzada de Tlalpan No. 3016 y 3058 Ex Hacienda Coapa, CP 04910 Coyoac, Mexico

**Keywords:** Public policy, México, Breast cancer

## Abstract

**Background:**

The recent increase of breast cancer mortality has put on alert to most countries in the region. However it has taken some time before breast cancer could be considered as a relevant problem. Only in recent years breast cancer has been considered a priority in some Latin American countries and resources have been mobilized to confront the problem at the institutional level. The article analyzes the efforts made in five Latin American countries (Argentina, Brazil, Colombia, Mexico and Venezuela) in the last 15 years to design and implement policies to address the growing incidence of breast cancer.

**Methods:**

Data was collected between July and December 2010 from both primary and secondary sources. Semi-structured interviews were conducted with key informants from governmental and non-governmental organizations. Secondary data was obtained from publications in journals, government reports and official statistics in each country. Analysis combines information from both types of sources.

**Results:**

Countries have followed different paths and are in different stages of policy implementation. In all cases early detection is a key strategy. Through the design of programs and guidelines, the allocation of financial resources to treat patients, as well as a formally structured information system, Brazil and Mexico have been able to set up comprehensive national policies. Argentina, Colombia and Venezuela have made important advancements but not yet capable of coordinating comprehensive national policies.

**Conclusion:**

Breast cancer is being considered a priority in all five countries but there are different stages in the rolling out of comprehensive national policies due to differences in their capacity to allocate resources, implement operational strategies and encourage the participation of relevant stakeholders.

## Background

Breast cancer mortality has been increasing significantly in the last decade in Latin America (LA). Epidemiological trends follow those identified in industrialized nations 20 years ago. Breast cancer is becoming an enormous challenge in developing countries together with diabetes, hypertension and obesity, among others [12, 19, 41].

Breast cancer mortality rate is increasing in most LA countries [14, 24, 26, 29] unlike cervical cancer that has been declining in recent years mainly due to the expansion of policies that promote –among other strategies- the dissemination of condoms as a barrier to the transmission of HPV, considered the infectious agent of cervical cancer [47, 51, 60]. However, breast cancer has not proved to be an infectious disease, thus demanding different coping strategies featuring the use of technologies and resources as well as behavioral changes based on promoting an active participation in screening programs along with the identification of emergency signs in order to trigger a care-seeking behavior [25, 61].

Breast cancer policies in the region normally share resources and are implemented jointly with those regarding cervical cancer. However the etiology of both diseases is completely different, moreover, it has been mentioned that their epidemiological trends also vary [50]. Unlike cervical cancer, breast cancer has not been specifically linked to just one cause, there are however a whole group of potential determinants. This feature makes it impossible to establish policies based on primary preventive actions. The most commonly used strategy is early detection which is considered to be a secondary preventive action [45].

Early detection is relevant because the earlier a cancer lesion is identified, the probability of survivorship increases, the invasive procedures can be avoided and the cost of treatment can be kept at a minimum [2, 7, 57]. In most industrialized countries early detection is guaranteed by screening female populations among age groups of 40–50 [16]. Mammography is the technology used to confirm initial diagnosis but the controversy about its efficacy is still open [27]. Nevertheless, industrialized countries have seen an important reduction in their mortality rates in recent years following a broad mammographic screening [6, 28].

Based on these facts, Latin American countries have started to develop governmental actions towards the implementation of a policy [17] to tackle the increase in breast cancer. It is important to assess the progress of recent developments heading towards the constitution of goal-oriented, structured and financed policies.

### Theoretical framework

According to Gilson [15] there are two main perspectives to define what health policy is about. First, the technical perspective that seeks to underpin the development of new policy or evaluates the result of a policy process in terms of the potential health benefits. Second, a more political and organizational approach that intends to understand the process in itself by identifying interested actors (individual, group or institutional) and their capacity to influence the policy process.

Those who adhere to the first perspective argue that it is possible to identify stages in the policy process while those who have a political perspective of the policy process do not assume that these stages are sequential or those they always occur in every decision-making process. Furthermore the second group tends to focus on the dynamics of human interaction, its modalities, and the role of hidden agendas and the capacity of individuals or groups to bias the process towards their particular interests.

Corkery, et al, [10] argue that the creation of a policy can be identified when incumbent actors, are capable of stating specific objectives that respond to a certain health problem. Incorporating the issue into the political agenda is the first step in turning it into a governmental priority. These objectives together with sound justifications and a normative frame have been incorporated into documents to be approved by legislative branches of government [1]. Most of these documents have a compulsory nature regulating the participation of interested parties. Normally policies derive into programs, guidelines and norms that specify the legal, administrative and technical nature of the activities to be carried out. Once policies are legally approved, they are frequently granted public resources to coordinate the implementation of concrete actions to accomplish the defined objectives. However, as initially mentioned, in most policy processes, these stages do not take place sequentially but they generally follow iterative patterns until final definitions are reached.

The analysis of breast cancer policies can be carried out based on the previous considerations but as in the majority of available policy analysis –including those interested in health issues- specificities should be considered to identify those key elements that characterize their essential nature [17]. In our case, a major consideration is that in Latin America and the Caribbean most breast cancer policies are actually being developed, clearly showing gaps in their technical and managerial definitions. A second consideration is that policies in the region are articulated based on the experience of European countries, USA and Canada but under different social, cultural and health conditions [58]. Third, they are framed in health systems that are being reformed aiming to develop stronger financial schemes, achieve universal coverage, increase the availability of resources (including information) and open up the system to the participation of more social actors [61, 62].

Existing breast cancer policies consider early detection as a key activity. However there are different ways to carry out this activity, which greatly depend on the structural characteristics of health care systems [3]. For example, health care systems have a better possibility of organizing successful screening programs when broad segments of the population have access to them.

Governmental entities are responsible for undertaking the stewardship role in the development and implementation of policies [5, 28]. Also major challenges emerge for the stewardship role when health systems are segmented or fragmented [39, 40]. Decentralization also implies a major challenge as local entities can identify their own priorities and implement their own policies, particularly when taxes are collected at that level [10]. In most cases, reference systems find enormous difficulties to operate due to the lack of organization, coordination and poor management [40].

In all stages of public policy, a diversity of actors are commonly involved. Policy is of course not only prerogative of public institutions but more recently following the increasing democratization in developing countries, civil society has emerged as a powerful actor [46] in its own right. In female cancers an important source of interest is the universal movement towards gender equity in health [53]. Civil society organizations (CSOs) currently participate in all stages of breast cancer policy. More recently CSOs and governmental entities seem to have found positive ways of interacting, which can promote that policies benefit targeted populations and not only interest groups which is always an inherent risk [4].

Most specialists convey that the generation of data and the analysis derived from it, is key in the policy cycle [22]. Data can be used to identify priorities, to draw up plans and programs and implement new operational mechanisms. Data can also be used to evaluate policy. Evaluating of policy is a key stage in identifying the achievement of defined goals. Moreover, data obtained from evaluations can be used to identify deviations in the policy process [52]. Data dissemination to interested parties is also a key democratic tool capable of informing society about the use of public resources and its consequences [43].

The following sections rely on a study carried out at the end of 2010 in five LA countries, Argentina, Brazil, Colombia, Mexico and Venezuela in order to assess the definition and implementation of breast cancer policy, the participation of CSOs in these endeavors and the capacity to set up routine data collection to evaluate and monitor the development of policy.

## Methods

The scarcity of information regarding breast cancer policies in LA [2, 17, 31, 44] motivated the development of a research project by a team of researchers from the National Institute of Public Health (NIPH) of Mexico between July and November of 2010 with support from the American Cancer Society.

The original study was based on a mixed-methods design. However, this article focuses only on the qualitative component that contains the core data used for the analysis of policies. Sample selection was intentional and carried out in two stages. In the first stage, countries were selected using the following criteria: a) structural diversity of health systems b) prevalence, incidence and mortality rates, c) variety of financing mechanisms, and d) participation of various stakeholders in the definition of breast cancer policy. In the second stage, key informants were selected from governmental and non-governmental organizations using the snow-ball technique [38, 54] from each of the selected countries (Table 1).

**Table 1:** Health Systems characteristics of five Latin American countries

Country	Total Population (thousands)	Health coverage^(a)^			Health systems type^(a)^	National health expenditure as a % of GDP		Physicians per 10,000 pop.
		Public	Social security	Private		Public	Private	
Argentina	41,119	30.2 %	60.8 %	9 %	Fragmented	6.2	3.2	Not available
Brasil	198,361	75 %	0 %	25 %	Unified	3.1	4.1	15.1
Colombia	47,551	Contributory regime: 39.7 %		Without insurance 4.3 %	Segmented and articulated	3.5	1.5	16.5
		Subsidized regime: 51.4 %						
		Special regimes: 4.6 %						
Mexico	116,147	36.6 %^(b)^	36.8 %^(b)^	0.44 %^(b)^ and without insurance 25.43 %	Segmented, not articulated	3.0	3.1	22
Venezuela (Boliviarian Republic of)	29,891	Not available	17.5 %^(c)^	11.7 %^(c)^ without insurance 68 %	Segmented, not articulated	Not available	2.4	Not available

*Source: Panamerican Health Organization (PAHO). Health situation in the Americas. Basic Indicators 2012. Available on:* http://ais.paho.org/chi/brochures/2012/BI_2012_ENG.pdf

^a^Pan American Health Organization. Health in the Americas: 2012 Edition. Regional Outlook and Country Profiles. Washington, DC: PAHO, 2012

^b^Gutiérrez JP, Hernández-Ávila M. Health protection coverage in Mexico and profile of unprotected population, 2000–2012. Salud Publica Mex 2013:55 suppl 2:S83–S90

^c^Bonvecchio A, Becerril-Montekio V, Carriedo-Lutzenkirchen A, Landaeta-Jiménez M. The health system of Venezuela. Salud Publica Mex 2011;53 suppl 2:S275–S286

Information was obtained by means of primary and secondary sources. In the first case, semi-structured interviews were applied to representatives from central and local government, CSOs’ personnel, professional associations’ representatives, and academics with experience in the field of breast cancer care. The final sample of informants was 65 in all five countries considered (Table 2).

**Table 2:** Informants by country

Type of informant	Mexico	Colombia	Venezuela	Brazil	Argentina	Total
Public sector	7	5	3	3	5	23
Civil society organization	3	5	8	3	3	22
Professional society	0^a^	1	3	3	1	8
Other stakeholder	4	2	4	1	1	12
Total	14	13	18	10	10	65

Source: own elaboration with data obtained from secondary sources

^a^In Mexico, it was not possible to interview an authorized representative of professional associations. However, a number of public sector respondents belonged to professional groups. An extended interview was carried out in these cases

Interviews were carried out in Spanish and Portuguese. A piloted semi-structured questionnaire was applied which facilitated the systematic exploration of topics. All informants provided a verbal informed consent before the interview. The main explored topics were the existence of national/local policies for breast cancer care, the implementation of policies through specific activities, the participant actors, resources available and finally, the attainment of objectives and goals.

Secondary sources gathered published and non-published official documents containing availability of physical, technological, human and financial resources to support the care of breast cancer as well as laws, guidelines, plans, programs, evaluation reports, clinical practice guidelines and articles addressing these topics (Table 3).

**Table 3:** Secondary information by country

Data source	Mexico	Colombia	Venezuela	Brazil	Argentina
Women’s health program	Yes	Yes	Yes	Yes	Yes
Sexual and reproductive health program	Yes	Yes	Yes	Yes	Yes
Breast cancer policies	Yes	Yes	No	Yes	Yes
Breast cancer care/control programs	Yes	No	Yes	Yes	Yes
Breast cancer treatment consensus	Yes	Yes	No	Yes	Yes
Breast cancer statistics (from national or regional surveys)	Yes	Yes	Yes	Yes	Yes
Breast cancer statistics (from continuous information system)	Yes	No	No	Yes	No

*Source*: Own elaboration with information from secondary sources

The processing of data from primary sources started with the transcription of audio recordings into Word format files. Data analysis (from primary and secondary sources) was guided through the design of codes organized by previously defined topics and sub-topics by the research team. A flexible approach was followed throughout the process in the integration and systematization of new information in order to identify emerging issues.

The project was approved by the Research and Ethics Committees of the National Institute of Public Health of Mexico # CI: 944, 944 September 27/2010.

## Results

Summarized in Table 4, results are presented in four sections as follows: a) definition of specific policy to confront breast cancer, b) early detection strategy, c) civil society participation, and d) information systems.

**Table 4:** Summary of basic epidemiological data and policy results by country

Country	Breast cancer epidemiology (Estimated incidence and mortality per 100,000 women 2008)^a^	Specific breast cancer policy	Early detection strategy	Civil society and civic participation	Information system
Mexico	Incidence: 27.2	Yes. Supported by a program with defined objectives and goals.	Organized opportunistic mammography screening program for women 40–69 years old.	High level of participation in the areas of education, research, service provision and lobbying but atomized.	Yes. SICAM PRO-MAMA is a routine data collection system.
	Mortality: 10.1				
Colombia	Incidence:31.2	No. Recently approved a cancer care law. National Cancer Institute is involved in policy.	Opportunistic mammography screening for both contributory and subsidized regimens. Not fully organized. Annual mammography starting at 49 years of age, except for symptomatic cases and the related risks.	High level of participation in the areas of education, research, service provision and lobbying but atomized.	No. Indicators produced through national surveys [35].
	Mortality: 10.0				
Venezuela	Incidence: 42.5	Not. The Ministry of Health has an under-funded sub-program for breast cancer control.	Sub-program with indications for opportunistic mammography screening carried out differently in each state. Starting age of mammography 35 years and for women with known risk factors at low age.	Low level of participation in program and policy definition. Active participation in educational activities and support for women with breast cancer.	Yes. Data collection is problematic and not all stakeholders are aware of its existence.
	Mortality: 13.7				
Brazil	Incidence:42.3	Yes. Supported by a program with defined objectives and goals.	Organized opportunistic mammography screening program for women 35 years and older and for women with known risk factors less than 35 years old.	High level of organized participation. Assigned seats in state and municipal health councils.	Yes. SISMAMA is a routine information collection system.
	Mortality: 12.3				
Argentina	Incidence: 74.0	Not at the national level. Some provinces have well developed policies.	No national screening program due to decentralized health system structure. In generally annual mammography starting from 49 years of age, except for symptomatic cases and the	Low level of participation in program and policy definition. Active participation in service provision.	No. Measured through national studies.
	Mortality: 20.1				

Source: own elaboration with information obtained from fieldwork primary and secondary sources

^a^Data [20]

### a) Definition of policy to confront breast cancer

The following elements were found among the group of countries to inform and characterize the development of breast cancer policy: 1) breast cancer as priority in the public health agenda, 2) allocation of specific funds to cover for breast cancer care, 3) the capacity of the central government to carry out and monitor the application of operational guidelines.

Even though breast cancer is identified as an important public health problem in all countries, only the Brazilian and Mexican health authorities have recognized it as a health priority. In these countries it has been incorporated as such in the public policy agenda ([32, 49]). In Argentina in spite of the fact that the rate of breast cancer mortality (21.8 per 100,000 women) is second in LA only behind Uruguay, various informants expressed that breast cancer is far away from being considered a public health priority. One of them pointed out:

*“Up to now, the government has said very little (about breast cancer policy).Plans were initiated and quickly disappeared. The current expectation is that the (new) National Cancer Institute could provide an assessment to understand the situation and see what we can do.* (Argentina 4)

Regarding financing, all breast cancer cases attended by the public system in Mexico and Brazil have guaranteed funds to cover all stages of the disease. In Venezuela, specific financing only exists for chemotherapy, which is managed by the Venezuelan Institute of Social Security. Argentina presents an opposite scenario considering that major problems were identified because breast cancer financing depends on the type of service package that different groups of populations are entitled to receive, generating major inequities in the possibility of these groups to have access to cancer care. In Colombia, before 2011, the scenario was similar to Argentina given the important differences between services packages provided to populations that were contributing to the social security fund and those who were fully subsidized by the state [55]. However, this scenario may have changed already –at least referring to treatment- following two legislative important changes: 1) the leveling of services packages between both types of populations [9], and 2) the definition of specific financing for breast cancer care within the social security fund [36].

Although all countries have programs, laws and/or clinical guidelines for breast cancer care, its implementation and operation is very lopsided both between and within each of the countries. In this sense all countries, with the exception of Venezuela, have set up National Cancer Institutes to carry out stewardship functions. However, the specific roles of Institutes differ according to the country. In Argentina, the new Cancer Institute created by presidential decree, is responsible for designing and guiding the implementation of a policy to combat different types of cancer, including breast cancer. The institute has been engaged, since its foundation, in negotiations with decentralized provinces for the definition of programs and specific guidelines for cancer care, a major objective in breast cancer is to set up a program for early detection. Brazil’s National Cancer Institute (INCA) has the dual function of guiding policy and providing hospital services to the population in the State of Rio de Janeiro. Its main specific function is to define national guidelines and try to negotiate its application in the more than 5,000 Brazilian municipalities. INCA also carries out research at different levels in order to produce evidence to back up its decisions. National Cancer Institutes in Mexico and Colombia share a specific focus on service provision. They were originally designed to provide hospital cancer care to the population. The Mexican institute also carries out clinical and epidemiological research. Both of them participate in the definition and monitoring of policy. Although neither of them is responsible for implementing policy, they do serve as advisory bodies to executive units.

### b) Early detection strategy

Early detection of breast cancer is a key activity in each country. However variations in its implementation can be identified.

In general, early detection policy calls for an annual or biannual mammography starting at age 50. However, in seeking to meet the specific needs of their at-risk populations, each country has adjusted the age group in which a mammography is to be initiated. Debate persists among government and non-government stakeholders on the appropriate age for initiating routine mammography [8, 11]. The increasing amount of women dying at young ages in LA has raised the possibility of lowering the age for a routine mammography initiation, as it was referred by a middle-level decision maker from the Mexican public health system:

“*In the year 2009, the updating process of the Breast Cancer Official Norm started. This process engaged into the discussion of lowering the age of the start of screening mammography down to 40 years, based on the existence of new epidemiological evidence that stated that breast cancer in Mexico was appearing at younger ages. In these discussion the main participants were the government, the academia and civil society organizations”*(Mexico 5)

In Brazil, a law was passed in 2009 to guarantee that every asymptomatic woman aged 35 or older would receive a mammography provided by public institutions on yearly basis and those who are under 35 if they present suspicious signs or have direct family history of breast cancer. In Venezuela, access to biannual mammography starts at age 35. In Mexico, the age for routine annual mammogram initiation (regardless of risk factor profile) was programmatically reduced after a two-year process of technical standard review.^1^ Colombia and Argentina preserve their initial definition of yearly mammography starting at 50, except for symptomatic and risk-related cases.

Nevertheless, early detection programs in all countries have major challenges to overcome. All of them follow an “opportunist” strategy where the possibility of detecting a woman with breast cancer depends on the probability that the person reaches the services which is unlike most European strategies that target all population at risk. Scarcity of resources and little awareness in the population have combined to produce poor results in early detection programs. In all countries the percentage of women who are detected at early stages is quite low [37, 48, 59]. In Brazil it has been estimated at 10 % [18]. Furthermore, inequities exist. In Argentina and Venezuela there is no national strategy of early detection and the possibility of having such a strategy depends on the province or state defining it as a priority and consequently allocating resources. In Mexico and Colombia women who are covered by the employees’ contributive sub-system have a higher chance of early detection compared to those financed and assisted by the public system. An informant from the Ministry of Health of Colombia stated the following:

*“There is an enormous gap in the timely detection of breast cancer due to the fragmentation of the health care system in two insurance modalities: the contributory and the subsidized. The first one has defined that it should reach 20 % of mammographic screening of its target population while the second has not established any goal at all”* (Colombia 5)

### c) Civil society and citizen’s participation

Civil society participation in Argentina and Venezuela is at the more spontaneous end of the spectrum. In Argentina various organizations have participated in promoting health activities for decades, though relationships with government institutions tend to be weak.

One exception is the Argentine League to Fight Cancer, which plays a distinct advisory role to health authorities regarding clinical care issues. In Venezuela the gap between health authorities and CSOs is so wide that the latter practically do not participate in any decisions related to breast cancer policy. In contrast, active dialogue to put pressure on government institutions was observed in Brazil, where CSOs have been incorporated into the structure of local government and state councils, making their voices significantly represented in many of the decisions made at different levels. One case to highlight is the Brazilian Federation of Philanthropic Institutions in Support to the Health of the Breast (FEMAMA) who has actively participated over a long period of time in the passing of the 2009 mammography norm. About this issue, one of the informants pointed out:

*“In 2002 conversations started to define a breast cancer policy. Once the analysis was completed in 2003, the ministry of health called upon the civil society and decision makers to debate about cancer policy”* (Brazil 3)

Key organizations have shown leverage in Mexico and Colombia and have successfully put pressure on health authorities when making decisions at the programmatic and policy level. CSOs have been particularly active in updating technical standards for breast cancer care in Mexico.

In all five countries, there are an increasing number of international stakeholders participating in various fronts. The pharmaceutical global corporate industry is participating using local CSOs as recipients of their support. For example, CIMA*B in Mexico and SenoSalud in Venezuela receive funds from companies to carry out their activities. Other non-pharmaceutical companies such as Avon, Caterpillar, General Motors, etc., also provide support to NGOs in order to carry out their advocacy roles. All corporations supporting CSOs obtain at least non-tangible benefits such as prestige and positive social image. Some CSOs are particularly successful in raising funds from various corporative sources in order to expand their capacity and influence. Not only are private for-profit businesses involved. The American Cancer Society (ACS), one of the most influential global cancer CSO supports local CSOs to carry out their advocacy roles through the provision of technical advice based on research evidence that ACS contracts. ACS has also supported the development of a regional organization called the Latin American Union for Breast Cancer (ULACCAM in Spanish) aiming at disseminating information throughout the region about participation strategies in the breast cancer arena. Another area of participation is through the implementation of clinical guidelines that have been drawn up to establish the basic conditions in providing breast cancer care. The Breast Global Health Initiative (BGHI) has developed a set of guidelines adapted to the needs of developing countries. BGHI has been trying to introduce its guidelines in LA with relative success. It is clear that international stakeholders have an important presence in the breast cancer field in all the studied countries.

### d) Information systems

Epidemiological information systems in Brazil and Mexico routinely produce information which allows prevalence, incidence and mortality to be estimated throughout the entire country. In contrast, Colombia, Argentina and Venezuela do not have national information systems that routinely collect information and are unable to monitor policy or program outcomes. However, alternative sources of information provide certain useful data for monitoring activities. In the words of a decision maker from the Venezuelan public health system:

*“Cancer registries have faced serious difficulties to have available enough human, material and financial resources to guarantee their continuous operation. These difficulties have affected the continuity of recordings, affecting quality and coverage targets*” (Venezuela 3)

A variety of structures and processes in the collection and use of information were observed. Brazil and Mexico have advanced the most in the implementation of specific information systems for different types of cancer. In Mexico, an information system for monitoring breast cancer care procedures has only recently been created, nevertheless, this step is crucial for policy monitoring at a time when breast cancer programs are in an expansion phase. In Brazil, INCA operates an information system that collects population based indicators as well as service provision indicators from all states and municipalities.

The way health care is financed and organized in Colombia substantially affects the collection and use of its information system. Organizations with health insurance functions called Management Companies Benefit Plans allocate funding to pay for health care and therefore have incentives to collect information on system users. While no mechanism currently exists to use information from these Entities for national policy monitoring, the National Cancer Institute is making a major effort to build an information system that meets these requirements. In Argentina the newly created Cancer Institute may help to overcome its information gaps in the coming years, as it is being depicted by the following testimony:

*“What the Institute of Cancer should do is to put together all registries (..) available throughout the country. We have registries in various provinces even in (small) localities such as Venados Blancos.”*(Argentina 4).

Table 4 summarizes a set of qualitative indicators used in the description of previous sections. Key information is included in each column to allow the situation of each of the five countries to be compared.

## Discussion

Although data collection showed that there is no specific sequence in the development of breast cancer policy stages in any of the countries, it was possible to identify four features that are common and relevant to provide a clear response to the breast cancer epidemics (definition of a specific policy, existence of an early detection strategy, civil society participation and development of information systems). However, there are important differences in the way they have developed in each country due to social and institutional traditions as well as the capacity of each country to allocate financial resources for their implementation. Although information obtained by the research was originally collected in 2010, the picture that is portrayed upon the description of the cases has not really changed in the last five years, except for Brazil. In this country a norm was recently approved [23] stating that no more than 60 days should pass between the diagnosis and the start of the treatment for all cancer patients. Furthermore, the public system is providing access to oral chemotherapy which allows patients in good physical conditions to receive treatment at home and, finally, Trastuzumab is now being provided to all breast cancer patients and is paid by the public system. However, over this period of time, the private sector dynamics in all countries including CSOs participation has to be highlighted allowing these organizations to increase their influence in designing and implementing these policies.

All five countries have made significant progress in ratifying national programs to combat breast cancer. The first feature has been to understand the health threat of breast cancer and a subsequent reaction from health authorities and interested stakeholders. The second important feature has been the definition of actions, plans and programs; and the third, implementation, which shows clear differences in progress by country [13, 30, 56].

Brazil and Mexico have made the greatest progress in both designing and operationalizing breast cancer policies. Both countries have official government documents that define the problem and describe actions to be carried out that are linked to specific goals [33, 49]. A sustainable, specific source of financing seems to be guaranteed in both countries as well. However, weak spots persist in day-to-day operations, mainly due to low coordination capacity. Defining breast cancer as a health priority in all Brazilian decentralized municipalities represents a major challenge, particularly when the prevalence in northern municipalities is quite low compared with that of the southern region, due mostly to socioeconomic and genetic factors [34]. Likewise, breast cancer mortality rates present differential regional patterns in Mexico (mortality is higher in the north than in the south), however the federal level of government appears to be more capable of standardizing its policy actions because of its centralized financing mechanism. Mexico’s main problem is a deficit of material and human resources [37].

In addition to regional differences in breast cancer magnitude, the dual health system in Colombia contributes to differentiate the service provision between the contributory and subsidized populations [55]. Although service packages have been recently equalized, it will take some time before historical differences in access to cancer care disappear both in ambulatory and hospital care. Argentina finds itself in a crucial phase in developing a breast cancer policy following the creation of its new National Cancer Institute, whose mission is to construct a harmonized health care policy for different types of cancer featuring breast cancer as one of its top priorities. While important progress has been made in the design of a policy, all provinces must be convinced to make a concerted effort. Finally, in Venezuela the most important obstacles in the consolidation of a coherent and functional breast cancer policy are the lack of coordination among different sectors and the persistence of inequities between rich and poor states.

Good policy harmonization always depends on the implementation of high-quality information. Despite important advances made in this area, it is imperative that countries create and/or strengthen their monitoring and evaluation systems. Mutual learning among LA countries on indicator design and technological implementation is a promising strategy that could drive progress in this area. Several voices have called about the need of better cancer registries to produce key data to feed clinical and policy process [21, 29, 42].

Finally, civil society plays a fundamental role in national efforts to combat breast cancer. CSOs must maintain their critical position but at the same time, be proactive regarding government-initiated proposals aiming at harmonizing the role of participating actors in the attainment of common goals. Breast cancer CSOs -many of them supported by global stakeholders- are gaining enormous power making them a formidable political driving force that could be used in favor of achieving public breast cancer goals.

## Conclusions

Breast cancer care is raising awareness among LA health authorities. This awareness is a result of both the increase in the death toll in women in the last ten years as well as the role of advocacy groups calling for a more decisive response from governments. The countries included in the study have already started actions to tackle breast cancer but not all cases have developed a structured policy. Actions differ according to the level of priority given in every country, the financial resources involved in it, the existence of a coordination entity at national level, the capacity to produce statistical data to guide decisions and the role of non-governmental actors (including civil society, professional groups, media and others). It is not possible to say at this point how successful analyzed countries will be in fully implementing breast cancer policies and reducing mortality in the long run but steps to accomplish that objective have been clearly identified in all of them.

## Abbreviations

ACS, American cancer society; BGHI, breast global health initiative; CSOs, civil society organizations; GN, Gustavo Nigenda; HPV, human papillomavirus; INCA, Brazil’s national cancer institute; LA, Latin America; LMGR, Luz Maria Gonzalez-Robledo; MCGR, Maria Cecilia Gonzalez-Robledo; RMBA, Rosa Maria Bejarano-Arias; ULACCAM, Latin American union for breast cancer; USA, United States of America

## Footnotes

1 The norm was revised in 2010. It now defines mammography at 40 every two years.
